# Supplementary material for: Perineal Wound Closure Following Abdominoperineal Resection and Pelvic Exenteration for Cancer: A Systematic Review and Meta-Analysis
Source: Cancers (Basel). 2021 Feb 10;13(4):721. doi: 10.3390/cancers13040721 (PMC7916499; doi:10.3390/cancers13040721)
Supplement: Supplementary file 1 [file cancers-13-00721-s001.pdf]

cancers

Supplementary Materials: Perineal Wound Closure Following Abdominoperineal Resection and Pelvic Exenteration for Cancer: A Systematic Review and Meta-Analysis

Etienne Buscail, Cindy Canivet, Jason Shourick, Elodie Chantalat, Nicolas Carrere, Jean-Pierre Duffas, Antoine Philis, Emilie Bérard, Louis Buscail, Laurent Ghouti and Benoit Chaput

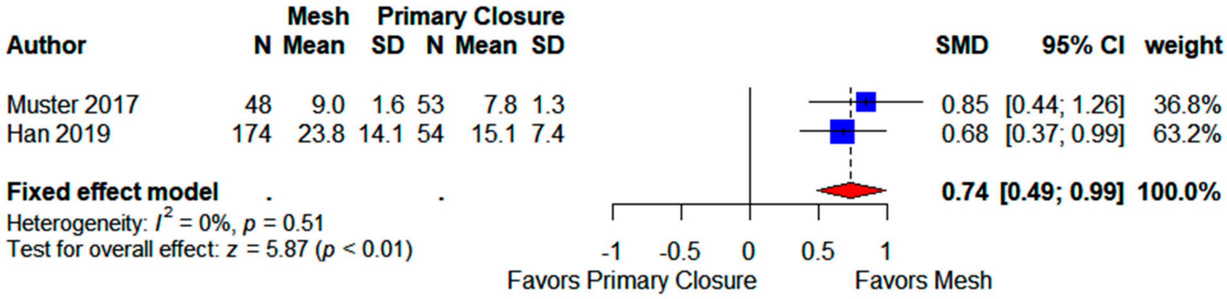

Figure S1. Forest plot for total lenght of stay mesh versus primary closure.

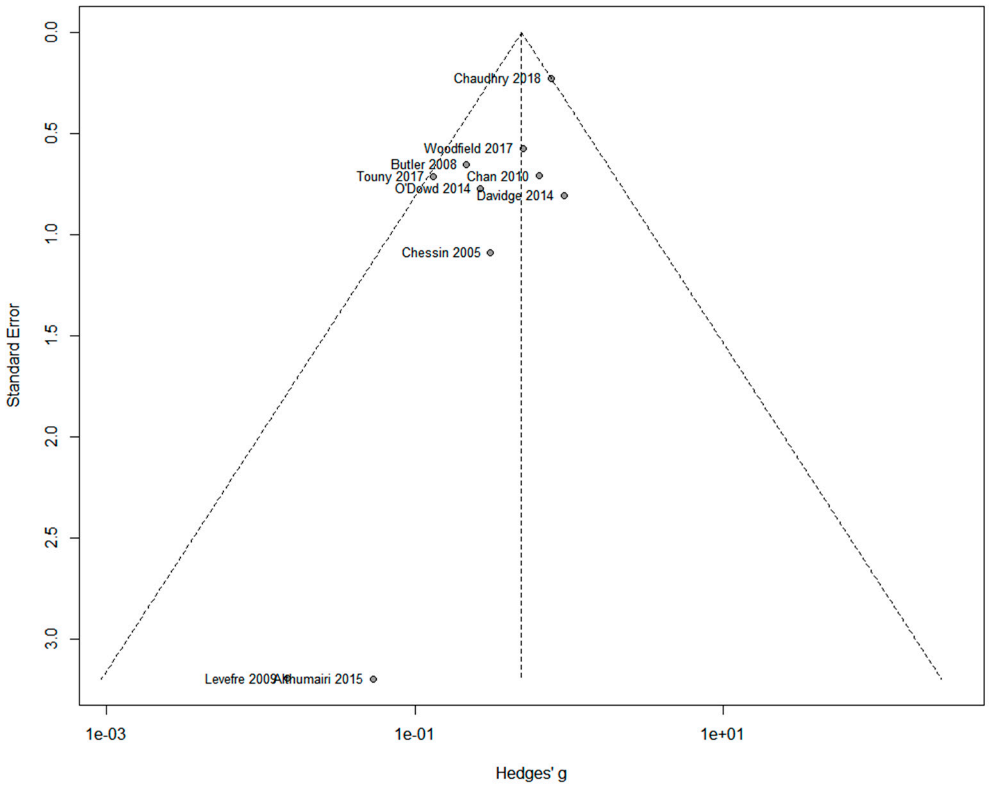

Figure S2. Funnel plot analyses for the detection of publication bias: flap vs primary closure group.

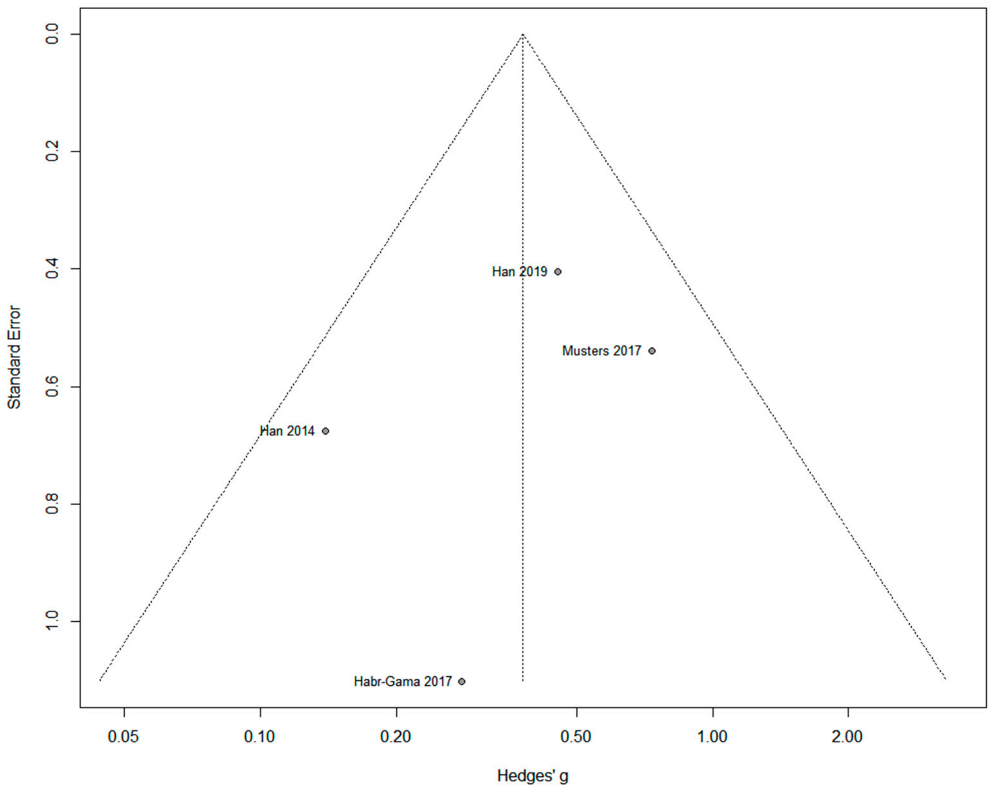

Figure S3. Funnel plot analyses for the detection of publication bias: mesh vs primary closure group.

Table S1. Electronic search strategy.

**Supplementary Table 1-1. Electronic Search Strategy in the PUBMED-Library and Cochrane Library**
Database: Medline via PubMed and Cochrane library<br>Filters: Publication date from 2000/01/01 and English<br>Initial search performed; September 6th 2019<br>Last rerun performed; March 28th 2020<br>Total hits: 2588
**Search Terms**
(abdominoperineal[tiab] OR abdominal perineal[tiab] OR APR[tiab] OR APER[tiab] OR ELAPE[tiab] OR proctectomy[tiab] OR abdominoperineal resection [tiab] OR abdominoperineal excision [tiab]
AND(Wound Healing [tiab] OR Surgical Wound Infection [tiab] OR Postoperative Complications[tiab] OR complication [tiab] OR morbidity [tiab] OR abscess [tiab] OR mortality[tiab] OR Wound [tiab] OR Healing [tiab] OR Reconstruction [tiab] OR Repair [tiab] OR flap [tiab] OR biological mesh [tiab] OR mesh [tiab] OR primary closure [tiab] OR length of stay [tiab] OR post-operative length of stay [tiab])

**Supplementary Table 1-2. Electronic Search Strategy in the EMBASE Library**
Database: EMBASE<br>Filters: Publication date from 2000/01/01 and English<br>Initial search performed; September 6th 2019<br>Last rerun performed; March 28th 2020<br>Total hits: 2588
**Search Terms**
(abdominoperineal OR abdominal perineal’ OR APR’ OR APER’ OR ELAPE’ OR proctectomy’ OR abdominoperineal resection ‘OR abdominoperineal excision ‘
AND (Wound Healing ‘OR Surgical Wound Infection ‘OR Postoperative Complications’ OR complication ‘OR morbidity ‘OR abscess ‘OR mortality’ OR Wound ‘OR Healing ‘OR Reconstruction ‘OR Repair ‘OR flap ‘OR biological mesh ‘OR mesh ‘OR primary closure ‘OR length of stay ‘OR post-operative length of stay ‘)

Table S2. Southampton wound assessment scale.

Grade	Definition	Appearance
0	Normal healing	
I	Normal healing with mild bruising or haematoma	A–some bruising<br>B–considerable bruising<br>C–mild erythema
II	Erythema plus other signs of inflammation	A–at one point<br>B–around sutures<br>C–along wound<br>D–around wound
III	Clear or haemoserous discharge	A–at one point only (<2 cm)<br>B–along wound (>2 cm)<br>C–large volume<br>D–prolonged (>3 days)
IV	Pus	A–at one point only (<2 cm) B–along wound (>2 cm)
V	Deep or severe wound infection with or without tissue breakdown; haematoma requiring aspiration	

Table S3. Newcastle Ottawa scale details.

Newcastle Ottawa scale					
**Study**	**Year**	**Selection**	**Comparability**	**Outcomes**	**Total**
Ghouti et al. [21]	2005	★★☆★	☆☆	☆★★	5
Chessin et al. [6]	2005	★★☆★	★☆	☆★☆	5
Butler et al. [7]	2008	★★☆★	★☆	☆★☆	5
Lefevre et al. [22]	2009	★★☆★	★★	☆★★	7
Chan et al. [23]	2010	★★☆★	☆☆	☆★☆	4
Stelzner et al. [24]	2011	★★☆★	☆☆	☆☆☆	3
Jacombs et al. [33]	2013	★★☆★	★☆	☆★☆	5
Chokshi et al. [32]	2013	★★☆★	★☆	☆★☆	5
O'Dowd et al. [27]	2014	★★★★	★☆	★★★	8
Touny et al. [28]	2014	★★★★	★★	★★★	9
Davidge et al. [25]	2014	★★☆★	☆☆	☆★☆	4
Han et al. [31]	2014	★★★★	★★	★★★	9
Althumairi et al. [26]	2015	★★☆★	☆☆	☆★☆	4
Woodfield et al. [29]	2015	★★☆★	☆☆	☆☆☆	3
Musters et al. [10]	2017	★★★★	★★	★★★	9
Habr-Gama et al [18].	2017	★★☆★	★☆	☆★★	6
Chaudhry et al. [30]	2018	★★☆★	★☆	☆★☆	5
Han et al. [9]	2019	★★☆★	★☆	☆★★	6

Table S4. Flap vs. primary closure: flap group general characteristics.

Study	Number of Patients *	Number of Patients Included in Flap Analysis	age Mean (Range)	Age Med (Range)	Rectal cancer	Anal cancer	Other indication	Neo-adjuvant RT short course	Neo-adjuvant RT long course	neo adjuvant CT
Ghouti 2005 [21]	41	26	NA	57.9 (32–82)	0	26	0	0	26	10
Chessin 2005 [6]	78	19	56.5 (32–74)	NA	12	7	0	0	19	19
Butler 2008 [7]	111	35	54 (SD 13.8)	NA	30	5	0	0	35	35
Lefevre 2009 [22]	95	43	56.3 (SD 11.9)	NA	0	43	0	0	43	43
Chan	51	30	NA	65.5	30	0	0	0	28	28

2010 [23]				(44-86)						
Stelzner 2011 [24]	74	28	NA	66 (44–79)	28	0	0	NA	NA	NA
Jacombs 2013 [33]	203	39	56.3 (SD 14.4)	NA	39	0	0	0	21	NA
Chokshi 2013 [32]	53	17	NA	62(38–72)	36*	2 *	15*	NA	13	NA
Davidge 2014 [25]	177	52	58.9 (SD 10.3)	NA	52	0	0	0	44	44
O'Dowd 2014 [27]	39	12	66.7 (SD 12.9)	NA	12	0	0	0	12	7
Touny 2014 [28]	60	30	NA	53.5 (26–68)	30	0	0	0	30	30
Althumairi 2015 [26]	67	11	57	NA	11	0	0	0	10	10
Woodfield 2017 [29]	68	31	63 (IQR18)	NA	12	9	10	NA	NA	NA
Chaudhry 2018 [30]	562	274	62	NA	NA	NA	NA	NA	134	75

* Entire cohort. SD, standard deviation, IQR interquartile range; NA, not available; RT, radiotherapy; CT, chemotherapy.

Table S5. Flap vs. primary closure: primary closure group characteristics general characteristics.

Study	Number of Patients *	Number of Patients Included in Primary Closure Analysis	age Mean (Range)	Age Med (Range)	Rectal Cancer	Anal Cancer	Other Indication	Neo-adjuvant RT Short Course	Neo-Adjuvant RT Long Course	Neo-Adjuvant CT
Ghouti 2005 [21]	41	15	NA	57.2 (32–82)	0	15	0	0	15	11
Chessin 2005 [6]	78	59	60.3 (25–80)	NA	56	3	0	0	59	32
Butler 2008 [7]	111	76	56.2 (SD 13.6)	NA	75	1	0	0	76	76
Lefevre 2009 [21]	95	52	62.1 (SD 13)	NA	0	52	0	0	52	52
Chan 2010 [23]	51	21	NA	67.8 (36–87)	21	0	0	0	13	13
Stelzner 2011 [24]	74	46	NA	64 (44–82)	46	0	0	NA	NA	NA
Jacombs 2013 [33]	203	164	60.6 (SD12.9)	NA	164	0	0	0	58	NA
Chokshi 2013 [32]	53	36	NA	55 (40–80)	36*	2 *	15*	NA	24	NA
Davidge 2014 [25]	177	125	59.8 (SD 13.4)	NA	125	0	0	0	106	106
	39	27	61.5	NA	27	0	0	0	20	17

O'Dowd 2014 [27]			(SD 11)							
Touny 2014 [28]	60	30	NA	51 (22–73)	30	0	0	0	30	30
Althumairi 2015 [26]	67	56	57	NA	56	0	0	0	43	45
Woodfield 2015 [29]	68	37	56	NA	23	1	13	NA	NA	NA
Chaudhry 2018 [30]	562	288	62	NA	NA	NA	NA	NA	127	72

* Entire cohort SD, standard deviation, IQR interquartile range; NA, not available; RT, radiotherapy; CT, chemotherapy.

Table S6. Mesh vs. primary closure: mesh closure group general characteristics.

Study	Number of Patients *	Number of Patients Included in Analysis	Age Mean	Age Med (Range)	Rectal Cancer	Anal Cancer	Other Cancer	Neo-Adjuvant RT Short Course	Neo-Adjuvant RT Long Course	Neo Adjuvant CT
Han 2014 [31]	102	83	NA	61 (27–78) *	83	0	0	83	0	83
Musters 2017 [10]	101	48	65 (SD 12)	NA	48	0	0	10	38	38
Habr-Gama 2017 [18]	72	22	NA	NA	22	0	0	0	22	22
Han 2019 [9]	228	174	NA	70 (42–80)	174	0	0	89	0	89

* Entire cohort SD, standard deviation, IQR interquartile range; NA, not available; RT, radiotherapy; CT, chemotherapy.

Table S7. Mesh vs. primary closure: primary closure group general characteristics.

Study	Number of Patients*	Number of Patients Included in Analysis	Age Mean (Range)	Age Med (Range)	Adenocarcinoma	Anal Cancer	Other Cancer	Neo-Adjuvant RT Short Course	Neo-adjuvant RT Long Course	Neo Adjuvant CT
Han 2014 [31]	102	19	NA	61 (27–78)	19	0	0	19	0	19
Musters 2017 [10]	101	53	64 (SD12)	NA	53	0	0	10	42	42
Habr-Gama 2017 [18]	72	48	NA	NA	48	0	0	0	48	48
Han 2019 [9]	228	54	NA	70 (27–80)	54	0	0	20	0	20

* Entire cohort SD, standard deviation, IQR interquartile range; NA, not available; RT, radiotherapy; CT, chemotherapy.

Table S8. Flap vs. primary closure: flap group surgical data.

Study	Number of Patients *	Number of Patients Included in Analysis	APR(n)	ELAPE(n)	Pelvic Exenteration	flap Failure(n)	Hernia(n)	Follow-Up (Month Med Range)

Ghouti 2005 [21]	41	26	0	26	0	3	0	67 (15–155)
Chessin 2005 [6]	78	19	19	0	0	0	NA	NA
Butler 2008 [7]	111	35	35	0	0	1	2	38.4 (SD27.6)
Lefevre 2009 [22]	95	43	0	43	0	0	0	25.9 (1.5–174.3)
Chan 2010 [23]	51	30	30	0	0	5	NA	17 (1–38)
Stelzner 2011 [24]	74	28	0	28	0	0	NA	NA
Jacombs 2013 [33]	203	39	0	0	39	9	NA	NA
Chokshi 2013 [32]	53	17	0	0	17	2	NA	11.5(1–60)
Davidge 2014 [25]	177	52	18	0	34	1	NA	17.2
O'Dowd 2014 [27]	39	12	12	0	0	0	0	18.2 primary SD 13.8 /15.2 SD 6.8
Touny 2014 [28]	60	30	30	0	0	0	NA	NA
Althumairi 2015 [26]	67	11	11	0	0	NA	NA	19.4
Woodfield 2017 [29]	68	31	31	0	0	2	2	NA
Chaudhry 2018 [30]	562	274	274	0	0	NA	10	26

* Entire cohort SD, standard deviation; IQR interquartile range; APR, abdominoperineal resection; ELAPE, extra-levator abdominoperineal excision; NA, not available.

Table S9. Flap vs. primary closure: primary closure group surgical data.

Study	Number of Patients *	Number of Patients Included in Analysis	APR (n)	ELAPE (n)	Pelvic Exenteration (n)	Hernia (n)	Follow-Up (Month Med Range)
Ghouti 2005 [21]	41	15	0	15	0	0	67 (15–155)
Chessin 2005 [6]	78	59	59	0	0	NA	NA
Butler 2008 [7]	111	76	76	0	0	4	50.4 (SD32.4)
Lefevre 2009 [22]	95	52	0	52	0	7	40.7 (1.5–174.3)
Chan 2010 [23]	51	21	21	0	0	NA	38 (16–58)
Stelzner 2011 [24]	74	46	46	0	0	NA	NA
Jacombs 2013 [33]	203	164	0	0	164	NA	NA
Chokshi 2013 [32]	53	36	0	0	36	NA	11.5 (1–60)
Davidge 2014 [25]	177	125	109	0	16	NA	14
O'Dowd 2014 [27]	39	27	27	0	0	0	16.8
Touny 2014 [28]	60	30	30	0	0	NA	NA

Althumairi 2015 [26]	67	56	56	0	0	NA	31.5
Woodfield 2015 [29]	68	37	37	0	0	0	NA
Chaudhry 2018 [30]	562	288	288	0	0	10	26

* Entire cohort SD, standard deviation; IQR interquartile range; APR, abdominoperineal resection; ELAPE, extra-levator abdominoperineal excision; NA, not available.

Table S10. Mesh vs primary closure: mesh closure group surgical data.

Study	Number of Patients *	Number of Patients Included in Analysis	APR(n)	ELAPE (n)	Hernia (n)	Length of Stay (day Med Range)	Follow-Up (Month Med Range)
Han 2014 [31]	102	83	0	83	4	10(7–62)	44(18–68)
Musters 2017 [10]	101	48	0	48	2	8(7–13)	12
Habr-Gama 2017 [18]	72	22	3	19	NA	NA	NA
Han 2019 [9]	228	174	0	174	7	13 (7–62)	71.5 (21–120)

* Entire cohort SD, standard deviation; IQR interquartile range; APR, abdominoperineal resection; ELAPE, extra-levator abdominoperineal excision.

Table S11. Mesh vs. primary closure: primary closure surgical data.

Study	Number of Patients*	Number of Patients Included in Analysis	APR(n)	ELAPE (n)	Hernia (n)	Length of Stay (Day Med Range)	Follow-Up (Month Med Range)
Han 2014 [31]	102	19	0	19	2	10 (7–62)	44 (18–68)
Musters 2017 [10]	101	53	0	53	4	7 (6–11)	12
Habr-Gama 2017 [18]	72	48	47	1	NA	NA	NA
Han 2019 [9]	228	54	0	54	6	11 (5–37)	58.5 (31–84)

* Entire cohort SD, standard deviation; IQR interquartile range; APR, abdominoperineal resection; ELAPE, extra-levator abdominoperineal excision; NA, not available.

Table S12. Flap vs. primary closure: Total wound complication.

Study	Flap Closure(n)	Number of Complications	% of Complication Flap Closure	Primary Closure (n)	Number of Complications	% of Complication Primary Closure
Ghouti 2005 [21]	15	11	73	26	17	65
Chessin 2005 [6]	19	16	84	59	44	74
Butler 2008 [7]	35	12	34	76	32	42
Lefevre 2009 [22]	41	11	25	43	19	36
Chan 2010 [23]	30	10	33	21	9	42
Stelzner 2011 [24]	28	3	10	46	8	17
Jacombs 2012 [33]	39	28	71	164	64	39
Chokshi 2013 [32]	17	13	76	36	26	72
Davidge 2014 [25]	52	28	54	125	42	34
O'Dowd 2014 [27]	12	3	25	27	15	55
Touny 2014 [28]	29	5	17	30	14	46
Althumairi 2015 [26]	11	3	27	56	24	42
Woodfield 2017 [29]	31	17	54	37	20	54
Chaudhry 2018 [30]	274	53	19	288	99	34

Table S13. Mesh vs. primary closure: Total wound complication.

Study	Mesh Closure(n)	Number of Complications	% of Complication Mesh	Primary Closure (n)	Number of Complications	% of Complication Primary
Han 2014 [31]	83	10	12	19	7	36
Musters 2017 [10]	48	16	33	53	17	32
Habr-Gama 2017 [18]	22	6	27	48	14	29
Han 2019 [9]	174	21	12	54	15	27

Table S14. Flap vs. primary closure: major wound complication.

Study	Flap Closure (n)	Number of Complications	% of Complication Flap Closure	Primary Closure (n)	Number of Complications	% of Complication Primary Closure
Chessin 2005 [6]	19	5	26	59	15	25
Butler 2008 [7]	35	3	8	76	23	30
Levefre 2009 [22]	41	0	0	43	6	13
Chan 2010 [23]	30	5	16	21	5	23
Jacombs 2012 [33]	39	10	25	164	23	14
Chokshi 2013 [32]	17	11	64	36	18	50
Davidge 2014 [25]	52	12	23	125	18	14
O'Dowd 2014 [27]	12	3	25	27	15	55
Touny 2017 [28]	29	3	10	30	14	46
Althumairi 2015 [26]	11	0	0	56	8	14
Woodfield 2017 [29]	31	6	19	37	12	32
Chaudhry 2018 [30]	274	41	14	288	54	18

Table S15. Mesh vs primary closure: Major wound complication.

Study	Mesh Closure	Number of Complications	% of Complication Mesh	Primary Closure	Number of Complications	% of Complication Primary
Han 2014 [31]	83	5	6	19	6	31
Musters 2017 [10]	48	7	14	53	10	18
Habr-Gama 2017 [18]	22	1	4	48	7	14
Han 2019 [9]	174	20	11	54	12	22

Table S16. Flap vs. primary closure: minor wound complication.

Study	Flap Closure (n)	Number of Complications	% of Complication Flap	Primary Closure (n)	Number of Complications	% of Complication primary
Chessin 2005 [6]	19	11	57	59	29	37
Butler2008 [7]	35	9	25	76	9	11
Lefevre 2009 [22]	41	11	26	43	13	30
Chan 2010 [23]	30	5	16	21	4	19
Jacombs 2012 [33]	39	25	64	164	22	13
Chokshi 2013 [32]	17	2	11	36	8	22
Davidge 2014 [25]	52	16	31	125	24	19
Touny 2014 [28]	29	3	10	30	10	33
Althumairi 2015 [26]	11	3	27	56	16	29
Woodfield 2017 [29]	31	11	35	37	8	21
Chaudhry 2018 [30]	274	12	4	288	45	15

Table S17. Mesh vs. primary closure: Major wound complication.

Study	Mesh Closure (n)	Number of Complications	% of Complication Mesh	Primary Closure (n)	Number of Complications	% of Complication Primary

Han 2014 [31]	83	5	6	19	1	5
Habr-Gama 2017 [18]	22	5	22	48	7	14
Musters 2017 [10]	48	9	18	53	7	13
Han 2019 [9]	174	1	0.5	54	3	5

Table S18. Follow up: Flap vs primary closure.

Study	Number Flap	Number Primary	Mean Follow Up*	Med Follow Up* (Range)
Ghouti 2005 [21]	15	26	NR	67 (15–155)
Chessin 2005 [6]	19	59	NR	NR
Butler 2008 [7]	35	76	38.4	NR
Lefevre 2009 [22]	43	52	49.7	NR
Chan 2010 [23]	30	21	17	18 (1–38)
Stelzner 2011 [24]	28	46	NR	NR
Jacombs 2012 [33]	39	164	NR	NR
Chokshi 2013 [32]	17	36	11.5	NR
Davidge 2014 [25]	52	125	24.4	NR
O'Dowd 2014 [27]	12	27	18.2	NR
Touny 2014 [28]	30	30	NR	NR
Althumairi 2015 [26]	11	56	31.5	NR
Woodfield 2017 [29]	31	37	NR	NR
Chaudhry 2018 [30]	274	288	26	NR

NR, not reported; SD, standard deviation; * entire cohort.

Table S19. Follow up: mesh vs. primary closure.

Study	Number Mesh	Number Primary	Mean Follow Up*	Med Follow Up (Range)*
Han 2014 [31]	83	19	NR	44 (18–68)
Musters 2017 [10]	48	53	12	NR
Habr-Gama 2017 [18]	22	48	14	NR
Han 2019 [9]	174	54	NR	71.5 (21–120)

NR, not reported; SD, standard deviation; * entire cohort.

Table S20. Length of stay: flap vs. primary closure.

Study	Number Flap Closure	Length of Stay Mean	SD	Length of Stay Med	Range Min	Range Max	Number Primary Closure	Length of Stay Mean	SD	Length of Stay Med	Range Min	Range Max
Ghouti 2005 [21]	15	25.2	8.5	25	12	40	26	27.3	16	22	12	80
Chessin 2005 [6]	19	11.3	NR	NR	NR	NR	59	12.2	NR	NR	NR	NR
Butler 2008 [7]	35	9.1	7.2	NR	NR	NR	76	10.2	3.8	NR	NR	NR
Lefevre 2009 [22]	43	25.1	10.7	NR	NR	NR	52	23.1	17.2	NR	NR	NR
O'Dowd 2014 [27]	12	19	13.2	NR	NR	NR	27	19.5	13.1	NR	NR	NR
Touny 2014 [28]	30	NR	NR	7	3	40	30	NR	NR	9	6	49
Althumairi 2015 [26]	11	13.4	9	NR	NR	NR	56	9.3	5	NR	NR	NR
Woodfield 2017 [29]	31	NR	NR	13	IQR 12	NR	37	NR	NR	13	IQR 8.5	NR

NR, not reported; SD, standard deviation; IQR, interquartile range.

Table S21. length of stay: mesh vs primary closure.

Study	Number Mesh Closure	Length of Stay Mean	SD	Length of Stay Med	Range Min	Range Max	Number Primary Closure	Length of Stay Mean	SD	Length of Stay Med	Range Min	Range Max
Muster 2017 [10]	48	NR	NR	8	7	13	53	NR	NR	7	6	11
Han 2019 [9]	174	NR	NR	13	7	62	54	NR	NR	11	5	33

NR, not reported.

© 2021 by the authors. Licensee MDPI, Basel, Switzerland. This article is an open access article distributed under the terms and conditions of the Creative Commons Attribution (CC BY) license (http://creativecommons.org/licenses/by/4.0/).
